# Deubiquitinase Genes as Prognostic Biomarkers in Osteosarcoma: Insights From Single-Cell Sequencing and Machine Learning–Based Prognostic Modeling

**DOI:** 10.1155/ijog/7932638

**Published:** 2025-08-17

**Authors:** Meng Zhang, Chunxin Cheng, Gang Liu

**Affiliations:** Department of Orthopedic, The Affiliated Huaian No. 1 People's Hospital of Nanjing Medical University, Huaian, Jiangsu, China

**Keywords:** deubiquitinase genes, prognostic model, single-cell RNA sequencing, tumor, tumor microenvironment

## Abstract

**Background:** Osteosarcoma (OS) is a highly aggressive bone tumor characterized by poor prognosis and frequent metastasis. Despite advances in treatment, the survival rate remains low. This study investigates the role of deubiquitinase (DUB) genes in OS to identify novel prognostic biomarkers and therapeutic targets.

**Methods:** We analyzed the expression of DUB genes using single-cell RNA sequencing (scRNA-seq) data from OS samples. Bulk RNA sequencing datasets were also assessed for survival correlation. A prognostic model was constructed by integrating DUB gene expression with clinical features using machine learning algorithms, including random forest and support vector machines. Immune cell infiltration in the tumor microenvironment (TME) was evaluated based on correlations between DUB gene expression and immune cell abundance.

**Results:** Single-cell analysis revealed significant heterogeneity in DUB gene expression across OS cell types, influencing tumor progression and cell–cell communication. The constructed prognostic model stratified OS patients into the high- and low-risk groups with significantly different survival outcomes. High-risk patients exhibited reduced immune cell infiltration and more aggressive clinical characteristics. The model was validated using three independent cohorts (TARGET-OS, GSE16091, and GSE21257), demonstrating high robustness and clinical utility.

**Conclusions:** DUB genes are critical regulators of OS progression and immune modulation. The developed prognostic model may serve as a promising tool for risk stratification and personalized treatment strategies in OS.

## 1. Introduction

Osteosarcoma (OS) is the most common primary malignant bone tumor in children and adolescents, representing a significant clinical challenge due to its high incidence and aggressive nature [1–3]. Characterized by substantial heterogeneity and a tendency to metastasize rapidly, OS remains difficult to treat despite advancements in treatment strategies, including surgical resection and chemotherapy [4–6]. Although survival rates have improved in recent decades, the prognosis for OS patients remains unsatisfactory, primarily due to the high rates of metastasis and recurrence [7–9]. Consequently, there is an urgent need for new, more effective biomarkers and therapeutic targets to refine prognostic stratification and improve treatment outcomes.

At the molecular level, post-translational modifications (PTMs) of proteins play a crucial role in regulating cellular functions [10, 11]. Among these, deubiquitination, a reversible process regulated by deubiquitinating enzymes (deubiquitinases (DUBs)), is of particular interest due to its involvement in key biological processes such as tumorigenesis, immune response modulation, and cell signaling [12–14]. DUBs remove ubiquitin from target proteins, thereby regulating protein stability, localization, and activity [15, 16]. Dysregulation of DUB activity has been implicated in the development and progression of various cancers, including OS [17]. Recent studies have demonstrated that dysregulation of DUBs contributes to oncogenic signaling, immune evasion, and therapy resistance in OS. For example, USP1 and USP7 have been implicated in DNA damage repair and tumor growth in OS [18, 19]. Meanwhile, the integration of multiomics data and machine learning algorithms offers promising avenues for building robust, individualized prognostic models [20]. Such approaches enable data-driven identification of clinically relevant biomarkers and have demonstrated superior performance compared to traditional methods. However, the precise role of DUB-related genes in OS remains largely unexplored.

In this study, we aim to comprehensively investigate the landscape of DUB genes in OS, focusing on their expression profiles at the single-cell level. By leveraging both single-cell RNA sequencing (scRNA-seq) and bulk transcriptome data, we seek to construct a robust prognostic model using machine learning approaches. Additionally, we explore the intricate relationship between DUB gene activity and the tumor microenvironment (TME), which plays a critical role in tumor progression, immune evasion, and therapeutic resistance. Through this integrative analysis, we identify key DUB-related signatures that not only provide valuable prognostic insights but also highlight potential therapeutic targets for OS. Ultimately, our findings may pave the way for the development of more personalized treatment strategies and improve clinical outcomes for OS patients.

## 2. Methods

### 2.1. Single-Cell Data Acquisition and Processing

scRNA-seq data (GSE162454) from six OS patients were downloaded from the GEO database [21–23]. Data processing was performed using the Seurat package. Cells expressing hemoglobin, mitochondrial, or ribosomal genes were excluded through quality control steps. Highly variable genes were selected for normalization and scaling. Batch effects across patients were corrected using Harmony. Dimensionality reduction was performed using t-SNE and UMAP algorithms. Although both t-SNE and UMAP were applied during initial dimensionality reduction, only t-SNE results were retained for visualization in this study due to better clustering clarity. Cell types were annotated based on canonical marker genes. Enrichment scores for DUB-related genes (Top 500 protein-coding genes from GeneCards, see Table S1) were calculated using the AUCell package. Cells were categorized into the DUB_high and DUB_low groups based on median scores, and CellChat was used to evaluate differences in intercellular communication between groups.

### 2.2. Bulk RNA-Seq Data Processing

We analyzed bulk transcriptomic data from three datasets: TARGET_OS (downloaded from GDC) and GSE16091 and GSE21257 (downloaded from GEO) [24, 25]. For TARGET_OS, RNA-Seq STAR-count data were converted to TPM, filtered for low-expression genes, and batch-corrected. For GEO datasets, data were retrieved using the GEOquery package, normalized using limma's normalizeBetweenArrays function, and probe IDs were mapped to gene symbols. All datasets were unified using standardized gene IDs, and batch effects were corrected using the sva package.

### 2.3. Prognostic Model Construction Based on DUB-Related Genes

Genes differentially expressed between the DUB_high and DUB_low groups and the Top 150 DUB score–correlated genes were used to construct prognostic models. We applied 101 machine learning algorithm combinations, including CoxBoost, LASSO, random survival forest (RSF), plsRcox, and SurvivalSVM. Model training was conducted using the TARGET_OS dataset with 10-fold cross-validation. Performance metrics (*C*-index, AUC, and Brier score) were used for evaluation. The best-performing model (CoxBoost + GBM) was validated using two independent cohorts (GSE16091 and GSE21257) to assess robustness and generalizability.

### 2.4. Model Validation and Performance Assessment

The prognostic performance of the final model was evaluated in both training and validation datasets. Kaplan–Meier (K-M) survival curves were plotted to compare the high-risk versus low-risk groups, and statistical significance was determined using the logrank test. Time-dependent ROC curves were generated at 1-, 3-, and 5-year time points to assess predictive accuracy. Principal component analysis (PCA) was used to visualize risk group separation. All analyses were performed using R 4.3.2, primarily employing the survival, timeROC, and ggplot2 packages.

### 2.5. TME and Immune Infiltration Analysis

In the TARGET dataset, immune infiltration levels were estimated using CIBERSORT, xCell, and MCP-counter. TME characteristics were evaluated using the ESTIMATE algorithm to calculate stromal score, immune score, and tumor purity. Differences between the high- and low-risk groups were assessed using the Wilcoxon rank sum test. Analyses were performed using the IOBR, ESTIMATE, and ggpubr R packages.

### 2.6. Gene Set Variation Analysis (GSVA) and Gene Set Enrichment Analysis (GSEA) for Functional Pathway Analysis

Functional enrichment analysis was performed in the TARGET_OS dataset. GSVA was conducted using the GSVA R package with hallmark gene sets from MSigDB, and group differences were analyzed using the Wilcoxon test. GSEA was conducted using the clusterProfiler package, with pathways ranked by log2 fold change and evaluated by permutation analysis. An FDR < 0.05 was considered statistically significant.

### 2.7. Statistical Analysis

All R package versions used are in Table S2. All statistical analyses were conducted using R software (v4.3.2). Comparisons between two groups were performed using the Wilcoxon rank sum test. K-M survival curves were compared using the logrank test. ROC and AUC values were calculated using the timeROC package. Correlation analysis was performed using Spearma's correlation. A *p* value < 0.05 was considered statistically significant unless otherwise stated.

## 3. Results

### 3.1. Single-Cell Sequencing Landscape of DUB Genes in OS

**Figure 1:**
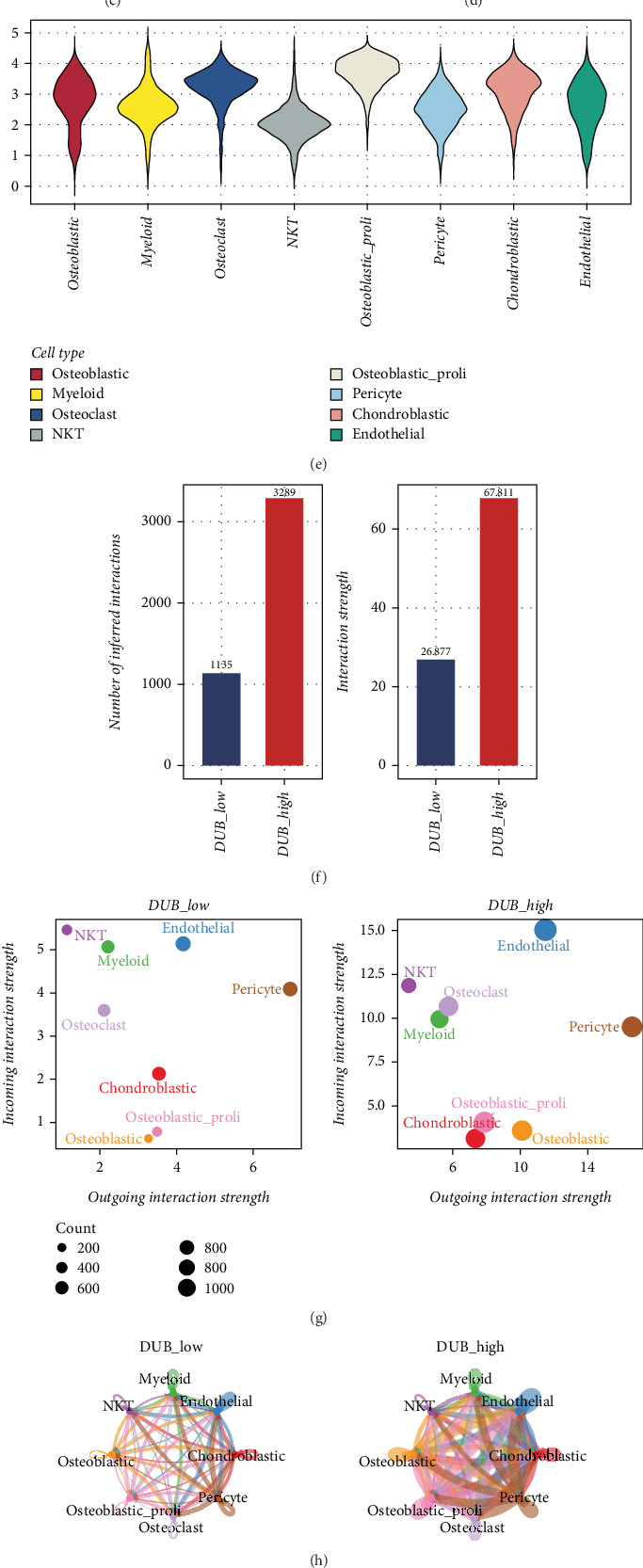
Single-cell sequencing landscape of DUB genes in osteosarcoma. (a) Dimensionality reduction and clustering of osteosarcoma single-cell data revealing 18 distinct cell clusters. (b) Cell type annotation based on marker genes, showing eight major cell types in osteosarcoma. (c) Proportional distribution of cell types across patient samples. (d, e) DUB gene enrichment scores for different cell types, with osteoblastic proliferation cells showing the highest DUB scores. (f) Cell–cell communication analysis showing significantly enhanced interactions in the DUB_high group. (g, h) Differential signaling interactions in the DUB_high group. (i) Correlation analysis of genes highly associated with the DUB score. (j) Signal flow analysis of cell–cell communication, confirming intensified signaling in the DUB_high group.

To investigate the single-cell sequencing landscape of DUB genes in OS, we first performed dimensionality reduction and clustering, identifying 18 distinct clusters (Figure 1a). Further cell annotation revealed eight major cell types, including osteoblastic cells, myeloid cells, osteoclasts, NK/T cells, osteoblastic proliferation cells, pericytes, chondrocytes, and endothelial cells (Figure 1b). The proportions of these cell types varied across patient samples, as shown in Figure 1c.

Next, we conducted enrichment scoring for DUB genes and found that osteoblastic proliferation cells exhibited the highest DUB score (Figure 1d,e). Based on the DUB score, all cells were classified into the DUB_low and DUB_high groups, followed by differential expression analysis. Cell–cell communication analysis revealed significantly enhanced intercellular interactions in the DUB_high group (Figure 1f), affecting both incoming and outgoing signaling (Figure 1g,h).

Furthermore, correlation analysis identified the Top 150 genes highly associated with the DUB score (Figure 1i). Signal flow analysis of cell–cell communication further confirmed that the DUB_high group exhibited markedly intensified signaling interactions (Figure 1j). These findings suggest that the deubiquitination process may play a crucial role in OS progression and intercellular communication.

### 3.2. Construction of an OS Prognostic Model Using 101 Machine Learning Algorithms and Their Combinations

**Figure 2:**
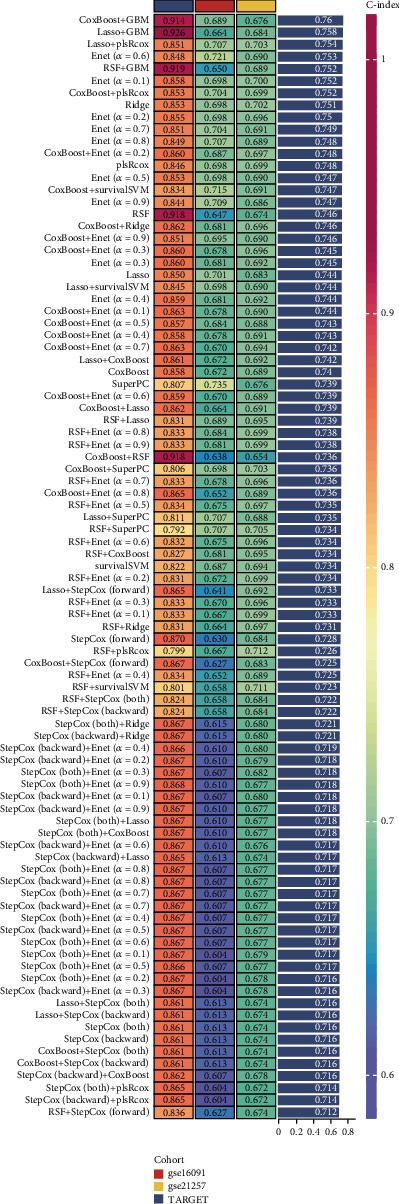
Prognostic model construction using machine learning algorithms: prognostic model construction using 101 machine learning algorithms, with the CoxBoost + GBM model showing the highest *C*-index of 0.76.

We constructed a prognostic model for OS using 101 machine learning algorithms and their combinations, with the TARGET_OS dataset as the training set and GSE16091 and GSE21257 as validation sets. Only models with high concordance index (*C*-index) values were presented. The results showed that the prognostic model derived from the CoxBoost + GBM algorithm achieved the highest average *C*-index of 0.76, making it the optimal model for further analysis (Figure 2).

### 3.3. Validation of the CoxBoost + GBM Prognostic Model

**Figure 3:**
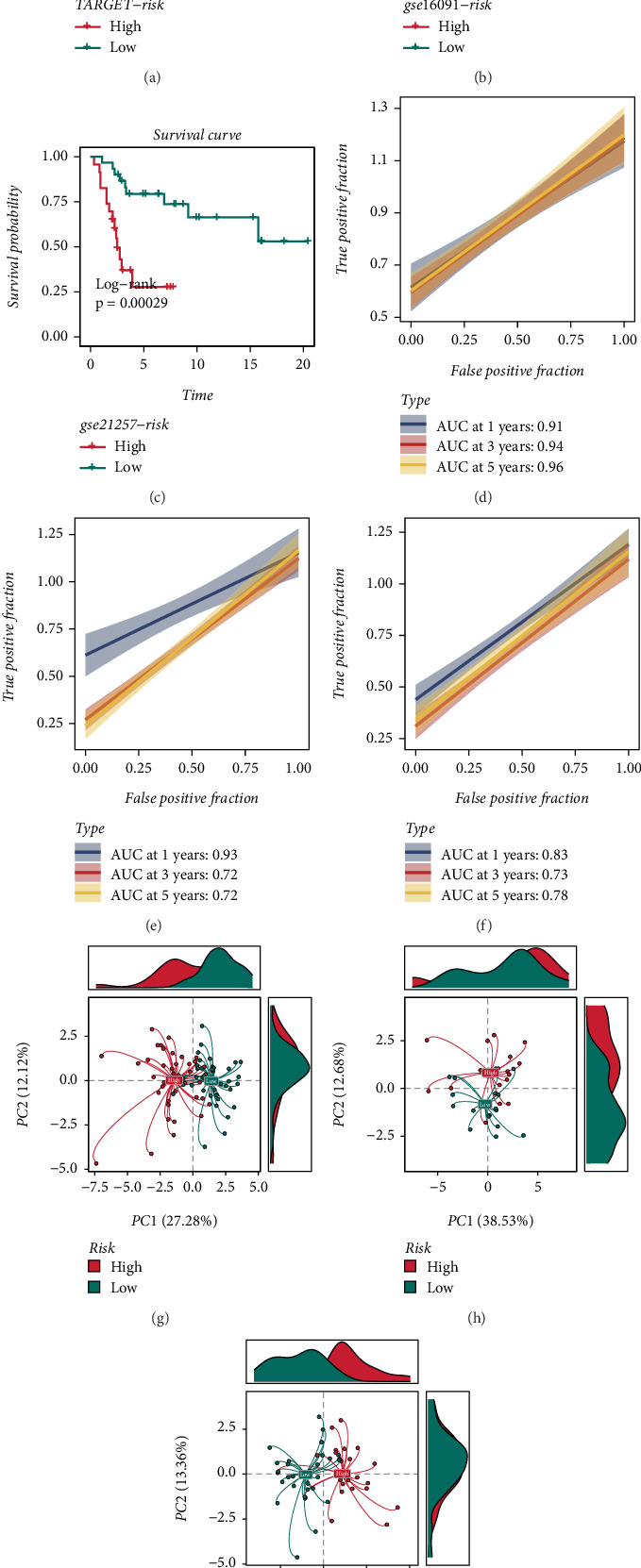
Validation of the CoxBoost + GBM prognostic model. (a–c) Kaplan–Meier survival curves comparing the high- and low-risk groups in the TARGET_OS, GSE16091, and GSE21257 datasets. (d–f) ROC curve analysis demonstrating the stable predictive performance of the CoxBoost + GBM model with AUC values above 0.8. (g–i) PCA plots showing the clear distinction between the high-risk and low-risk groups across datasets.

To evaluate the robustness of the CoxBoost + GBM prognostic model, K-M survival curves were generated for the TARGET_OS, GSE16091, and GSE21257 datasets. Patients were divided into the high-risk and low-risk groups using the median risk score as the cutoff. The results demonstrated that patients classified into the high-risk group had significantly worse prognoses than those in the low-risk group (Figures 3a, 3b, and 3c).

Furthermore, receiver operating characteristic (ROC) curve analysis indicated that the CoxBoost + GBM model maintained stable predictive performance across all three cohorts, with 1-year AUC values exceeding 0.8 (Figures 3d, 3e, and 3f). PCA further confirmed the strong discriminative ability of the CoxBoost + GBM model in distinguishing between risk groups in TARGET_OS, GSE16091, and GSE21257 (Figures 3g, 3h, and 3i).

### 3.4. TME Analysis

**Figure 4:**
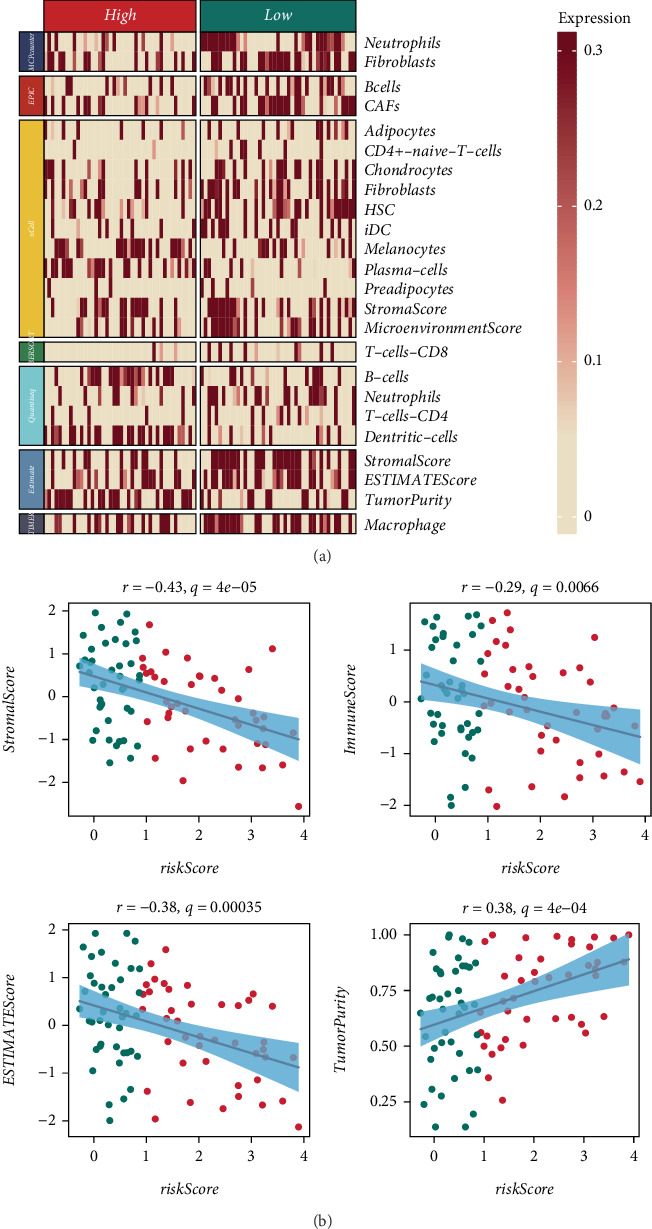
Tumor microenvironment (TME) analysis. (a) Immune cell infiltration analysis showing significantly lower immune infiltration in the high-risk group. (b) TME scoring analysis indicating negative correlations between risk scores and stromal/immune scores and a positive correlation with tumor purity.

Next, we evaluated the role of the CoxBoost + GBM model in the TME within the TARGET_OS cohort. Immune infiltration analysis revealed that immune cell infiltration was significantly lower in the high-risk group, which may be associated with a poor prognosis (Figure 4a).

Further TME scoring analysis demonstrated that the risk score was significantly negatively correlated with stromal score, immune score, and estimate score, while showing a significant positive correlation with tumor purity (Figure 4b).

### 3.5. GSEA

**Figure 5:**
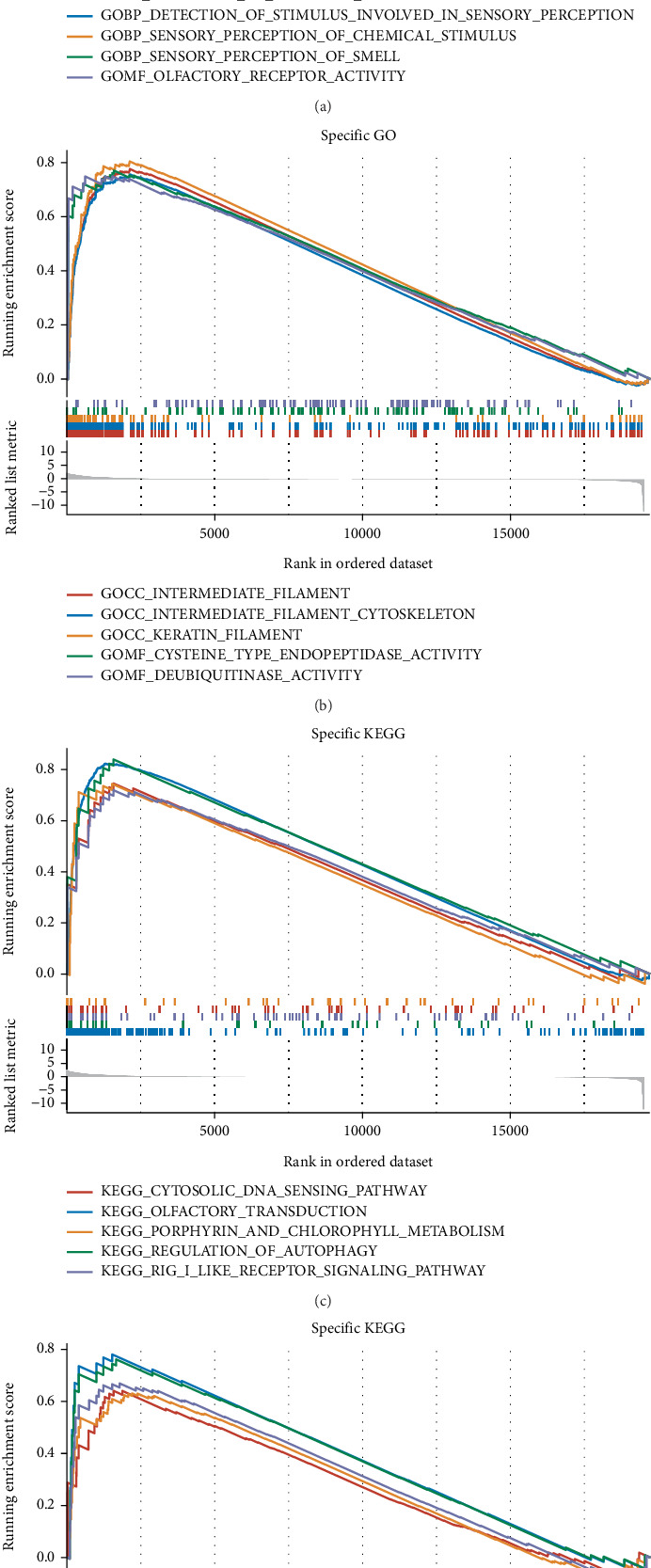
GSEA of signaling pathways. (a–d) Gene set enrichment analysis (GSEA) of the high-risk vs. low-risk groups (based on the CoxBoost + GBM model) using the TARGET-OS dataset. Significantly enriched pathways include response to chemical stimulus, intermediate filaments, deubiquitination, and cytosolic DNA sensing pathways. *p* values < 0.05 and FDR < 0.25 were considered statistically significant.

To further investigate the differences in signaling pathways between the high- and low-risk groups, we performed GSEA. Gene Ontology (GO) enrichment analysis revealed that the high-risk group was significantly enriched in pathways related to response to chemical stimulus, intermediate filaments, and deubiquitination. Meanwhile, KEGG enrichment analysis indicated significant enrichment of the cytosolic DNA sensing pathway in the high-risk group (Figures 5a, 5b, 5c, and 5d).

### 3.6. GSVA of Immune-Related Pathways

To investigate immune-related pathways in OS, we performed GSVA on the TARGET dataset to compute enrichment scores for immune-related pathways. First, we loaded the expression profile data and calculated the immune pathway enrichment score for each sample.

**Figure 6:**
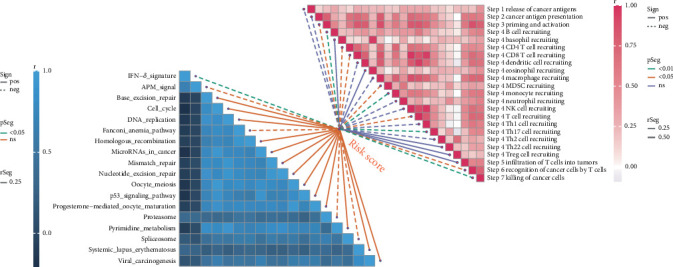
GSVA of immune-related pathways. GSVA was conducted using the “GSVA” R package on bulk RNA-seq data from the TARGET-OS dataset. Immune pathways were negatively correlated with risk scores, while cell cycle and proliferation-related pathways were positively correlated. Pearson correlation was used, with *p* values < 0.05 considered significant.

The results revealed that the risk score was negatively correlated with multiple immune pathways but positively correlated with pathways related to cell proliferation (Figure 6). These findings suggest that the risk score may be positively associated with OS progression.

### 3.7. Identification of the Key Gene BNIP3

**Figure 7:**
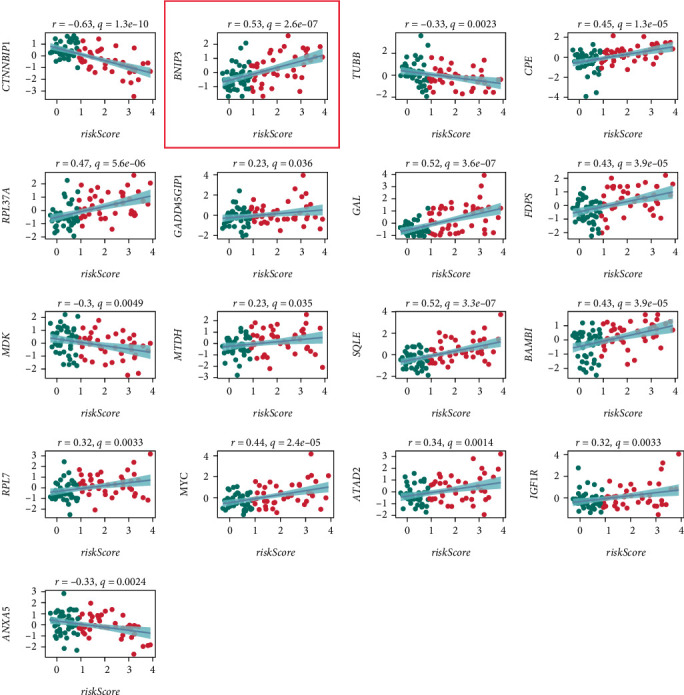
Identification of the key gene BNIP3.

Further analysis of the prognostic model revealed that it consisted of 17 genes. Among them, genes with positive coefficients (coefs) included BNIP3, CPE, RPL37A, GADD45GIP1, GAL, FDPS, MTDH, SQLE, BAMBI, RPL7, MYC, ATAD2, and IGF1R, while those with negative coefs included CTNNBIP1, TUBB, MDK, and ANXA5 (Figure 7). Among these genes, BNIP3 exhibited a strong positive correlation with the risk score, leading to its identification as a key gene potentially involved in OS pathogenesis.

Correlation analysis of key prognostic genes revealed that BNIP3 expression showed a strong positive association with the risk score. Data derived from the TARGET_OS cohort. Spearman's correlation coef was used for analysis.

### 3.8. BNIP3 Is Highly Expressed in OS Samples

**Figure 8:**
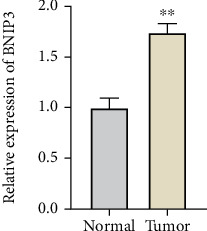
Validation of BNIP3 mRNA expression in clinical samples.

To validate the expression level of BNIP3 identified in our prognostic model, we performed quantitative real-time PCR (qRT-PCR) on OS samples. As shown in Figure 8 (⁣^∗∗^*p* < 0.01), BNIP3 mRNA was significantly upregulated in the tumor group compared to normal controls, consistent with our model predictions and public datasets.

## 4. Discussion

In this study, we systematically investigated the single-cell transcriptomic landscape of DUB genes in OS and developed a robust prognostic model using machine learning. Our findings provide novel insights into the role of deubiquitination in OS progression and its potential implications for prognosis and therapeutic targeting.

Our single-cell analysis revealed distinct cellular heterogeneity in OS, identifying eight major cell types. Notably, osteoblastic proliferative cells exhibited the highest DUB scores, suggesting a potential role for deubiquitination in OS cell proliferation. Furthermore, differential cell–cell communication analysis demonstrated enhanced intercellular interactions in the DUB_high group, implicating deubiquitination in TME modulation. These findings highlight the importance of DUB-related pathways in OS pathophysiology.

By leveraging machine learning techniques, we constructed a prognostic model based on DUB-related gene signatures. Among the 101 tested algorithms, the CoxBoost + GBM model achieved the highest predictive performance, demonstrating robust prognostic capabilities across multiple independent cohorts. The K-M survival analysis, time-dependent ROC curves, and PCA confirmed the model's ability to stratify OS patients into distinct risk groups with significant survival differences. These results underscore the prognostic value of DUB-related genes and their potential clinical application in risk stratification.

Furthermore, TME analysis revealed significant differences in immune cell infiltration between the high-risk and low-risk groups. Immune infiltration scores, including stromal and immune scores, were markedly lower in high-risk patients, while tumor purity was significantly higher. These observations suggest that high-risk OS patients may exhibit an immunosuppressive microenvironment, which could contribute to poor prognosis. Consistent with these findings, GSVA and GSEA demonstrated that immune-related pathways were significantly downregulated in high-risk patients, further supporting the notion that immune evasion mechanisms may be driven by deubiquitination processes in OS.

Additionally, specific DUB genes were found to be significantly associated with immune checkpoint molecules, indicating that targeting DUB pathways may enhance the efficacy of immune checkpoint inhibitors. This observation suggests a potential therapeutic strategy that combines DUB inhibitors with immunotherapy to overcome immune resistance in OS patients. Future studies should focus on validating these findings through experimental and clinical research to establish the feasibility of DUB-targeted therapies.

At the mechanistic level, deubiquitination may influence OS progression by regulating key oncogenic and tumor-suppressive pathways. For example, certain DUBs have been implicated in stabilizing proteins involved in the PI3K/AKT and Wnt/*β*-catenin signaling pathways, both of which are critical for OS cell survival and metastasis [26]. Understanding how DUBs modulate these pathways could reveal novel intervention points for therapeutic development.

From a clinical perspective, the identification of DUB-related signatures holds promise for personalized medicine approaches. Patients with high DUB activity may benefit from targeted therapies aimed at modulating deubiquitination, while those with lower DUB scores could be stratified for alternative treatment strategies. Moreover, integrating DUB-related biomarkers with existing prognostic factors may improve risk assessment and guide therapeutic decision-making.

In the model we constructed, BNIP3 is the key gene. The high expression of BNIP3 in OS was further confirmed by our qRT-PCR experiments, suggesting its potential as a biomarker. BNIP3 is a hypoxia-inducible protein that plays a dual role in cell death and autophagy [27]. Its overexpression has been linked to increased resistance to apoptosis and immune evasion under hypoxic conditions. In OS, recent studies suggest that BNIP3 may contribute to tumor aggressiveness by promoting a hypoxia-tolerant TME, thus representing a potential target for therapeutic intervention [28]. Similarly, MYC is a classic oncogene involved in cell proliferation, and IGF1R is implicated in OS growth and resistance to therapy [29, 30].

Taken together, our findings highlight the crucial role of deubiquitination in OS progression, immune regulation, and therapy resistance. The integration of single-cell and bulk transcriptome data enabled the identification of key DUB-related genes with prognostic significance. Future studies should focus on elucidating the mechanistic links between DUB activity and tumor immune evasion, as well as exploring potential therapeutic strategies targeting DUBs to enhance antitumor immunity.

## Data Availability

The datasets generated and/or analyzed during the current study are available in the Gene Expression Omnibus (GEO) repository, GSE16091 and GSE21257.

## Supporting Information

Supporting Information 1 Table S1: The top 500 deubiquitination genes in GeneCards.

Supporting Information 2 Table S2: R package and tools version.
