# Supplementary material for: Deubiquitinase Genes as Prognostic Biomarkers in Osteosarcoma: Insights From Single-Cell Sequencing and Machine Learning–Based Prognostic Modeling
Source: Int J Genomics. 2025 Aug 17;2025:7932638. doi: 10.1155/ijog/7932638 (PMC12375863; doi:10.1155/ijog/7932638)
Supplement: Supporting Information 2 — Table S2: R package and tools version. [file 7932638.f2.docx]

Supplemental Table 2. R package and tools version.

| **Tool/Package** | **Version** | **Application** | **Parameters / Notes** |
| --- | --- | --- | --- |
| **Seurat** | 4.3.2 | scRNA-seq processing, QC, clustering | VariableFeatures, FindClusters, RunUMAP |
| **Harmony** | 1.0 | Batch effect correction in scRNA-seq | Default parameters |
| **AUCell** | 1.16.0 | Scoring of DUB gene enrichment | AUC threshold: default |
| **CellChat** | 1.6.1 | Cell–cell communication analysis | identifyOverExpressedGenes, computeCommunProb |
| **limma** | 3.52.1 | Bulk data normalization and differential expression | normalizeBetweenArrays |
| **sva** | 3.44.0 | Batch effect correction for expression matrix | ComBat |
| **GEOquery** | 2.66.0 | GEO data download | Series Matrix |
| **GSVA** | 1.44.0 | Gene set variation analysis | msigdb.v7.5.1 symbols |
| **clusterProfiler** | 4.6.0 | GSEA pathway enrichment | FDR < 0.05 |
| **survival** | 3.5-5 | Survival analysis and Cox models | Kaplan-Meier, log-rank |
| **timeROC** | 0.4 | Time-dependent ROC curve | 1-, 3-, 5-year |
| **ggplot2** | 3.4.4 | Visualization | Survival and PCA plots |
| **IOBR** | 0.99.9 | Immune inference and TME analysis | Combined algorithms: CIBERSORT, xCell, MCP |
| **ESTIMATE** | 1.0.13 | Stromal, immune, and tumor purity scores | default |
